# Ensemble of global climate simulations for temperature in historical, 1.5 °C and 2.0 °C scenarios from HadAM4

**DOI:** 10.1038/s41597-024-03400-2

**Published:** 2024-06-04

**Authors:** Jesús Lizana, Nicole D. Miranda, Sarah N. Sparrow, Peter A. G. Watson, Miriam Zachau Walker, David C. H. Wallom, Malcolm D. McCulloch

**Affiliations:** 1 Future of Cooling Programme, Oxford Martin School, University of Oxford, Oxford, OX1 3BD UK; 2 Energy and Power Group, Department of Engineering Science, University of Oxford, Parks Road, Oxford OX1 3PJ UK; 3 Oxford e-Research Centre, University of Oxford, Oxford, OX1 3QG UK; 4 School of Geographical Sciences, University of Bristol, Bristol, BS8 1SS UK

**Keywords:** Climate-change impacts, Environmental impact, Climate sciences, Environmental sciences

## Abstract

Large ensembles of global temperature are provided for three climate scenarios: historical (2006–16), 1.5 °C and 2.0 °C above pre-industrial levels. Each scenario has 700 members (70 simulations per year for ten years) of 6-hourly mean temperatures at a resolution of 0.833° ´ 0.556° (longitude ´ latitude) over the land surface. The data was generated using the climate*prediction*.net (CPDN) climate simulation environment, to run HadAM4 Atmosphere-only General Circulation Model (AGCM) from the UK Met Office Hadley Centre. Biases in simulated temperature were identified and corrected using quantile mapping with reference temperature data from ERA5. The data is stored within the UK Natural and Environmental Research Council Centre for Environmental Data Analysis repository as NetCDF V4 files.

## Background & Summary

This dataset corresponds to the expected worldwide temperatures for a historical (2006–16) and two simulated scenarios widely referenced by the Intergovernmental Panel for Climate Change (IPCC)^1–3^: 1.5 °C and 2.0 °C of global mean temperature rise above pre-industrial levels.

Previous works^4–8^ use mean daily temperatures with different spatial resolutions to understand the impact of climate change projections. This work presents, for the first time, a 6-hourly temporal resolution for historical and two warming scenarios (1.5 °C and 2.0 °C) at a spatial resolution of 0.883° × 0.556° over the global land surface. The ensemble size is the largest for temperature found at this spatiotemporal resolution. The novelty lies in the combination of three factors: large ensemble size (700 members: 70 simulations for ten years), high spatiotemporal resolution (6-hourly mean temperatures at 0.833°´0.556°), and the representation of global mean temperature rise scenarios for 1.5 °C and 2.0 °C globally regardless of when these occur. The higher resolution compared to other large ensemble datasets also results in a better representation of weather phenomena, particularly dynamic situations such as blocking and jet stream variability^9^.

This data is the foundation for identifying the world locations that will be most affected by climate change in terms of temperature under two important IPCC scenarios. Its uses can be extended to estimate the impact of climate change on worldwide demand for cooling and heating, as well as climate zones, amongst others.

## Methods

This section describes the method used to generate the dataset, including the experimental framework and methods to process the raw results from the simulations.

### Data generation method

Ensembles of global climate simulations for mean temperature for three scenarios were generated using the climate*prediction*.net (CPDN) climate simulation environment^10,11^ to run the HadAM4 Atmosphere-only General Circulation Model^11,12^ (AGCM) from the UK Met Office Hadley Centre. The model’s horizontal resolution is 0.833° longitude and 0.556° latitude. The three scenarios examined represent mean temperatures at a historical period (2006–16) and 10-year periods with a global mean temperature rise of 1.5 °C and 2 °C above pre-industrial levels, regardless of when these occur. Each scenario has 700 members (70 simulations for ten years) with a temporal resolution of 6 hours and a spatial resolution of 0.833° longitude and 0.556° latitude.

These proposed scenarios follow the half-a-degree additional warming prognosis and projected impacts (HAPPI) experimental design protocol^13^. For the historical period, the sea surface temperatures (SSTs) and sea ice as boundary conditions for the atmospheric model are taken from the Operational SST and Ice Analysis^14^. Sulphur dioxide (SO_2_), dimethyl sulphide emissions, and ozone concentrations were specified following the Representative Concentration Pathways (RCP) 4.5 scenario. The ozone concentrations were zonally symmetric. For the 1.5 °C and 2 °C global warming scenario simulations, the SSTs, sea ice, greenhouse gas emissions and SO_2_ emissions were perturbed following Mitchell *et al*.^13^. Ozone is specified in both scenarios as a repeating annual cycle of the 2095 values of the RCP2.6 scenario. Solar and volcanic forcings followed those in Guillod *et al*.^15^ for 2091–2100.

The simulation experiments are run within CPDN^10,11^ using the Berkeley Open Infrastructure for Network Computing (BOINC)^16^ framework to distribute a large number of individual computational tasks. This system utilises the computational power of publicly volunteered computers. The process is described by Schaller *et al*.^17^, with the difference that (due to the higher resolution model used here), the long simulations performed to spin up the model with the imposed boundary conditions were not done on public computers. Instead, the spin-up used high-performance computing, with simulations ran for the full 10-year lengths of each historical and 1.5 °C and 2 °C global warming scenarios. Ten spin-up ensemble members were run for each scenario to achieve variability in the spin-up conditions. To create initial conditions for the main CPDN runs, for each start date, which occurred on the first of given months, model restart files were sampled every 5 days between the fifteenth of the previous month to fourteen days after the start date (e.g. October 15th to November 15^th^, 2006 for a November 1^st^, 2006 start). This model’s initial condition files provided 70 restart files per start date. The simulations in CPDN were initialised on November 1^st^, February 1^st^, May 1^st^, and August 1^st^. To further increase the ensemble size, additional perturbations were applied to the potential temperature field of the starting conditions. Potential temperature perturbations were calculated from 1-day differences within 15 days of the initialisation month and day from all years of the spin-up simulation. The initial month of each simulation was discarded as an adjustment period to these perturbations, similar to previous studies^13,15,17^.

The ensemble members per scenario were generated and obtained into four batches per scenario, detailed in Table 1. The members per batch were collected using extraction scripts (available at: 10.5281/zenodo.7573193). Data extraction and filtering ensured the same number of members per batch, year, and scenario. They randomly filtered 70 runs for each year. As a result, considering the 10-year period of each scenario, a total of 700 annual simulations were compiled: historical (April 2006 to March 2016), 1.5 °C and 2 °C.

**Table 1:** Summary of data structure by scenario and seasons^*^.

Scenario	Winter December-March	Spring April-May	Summer June-September	Autumn October-November
Historical (2006–16)	Batch 889	Batch 920	Batch 901	Batch 923
1.5 °C^**^	Batch 891	Batch 921	Batch 902	Batch 924
2.0 °C^**^	Batch 895	Batch 922	Batch 903	Batch 925

^*^ Seasons are used here as four time periods according to the Northern Hemisphere.

^**^The model was forced to increase global mean temperature according to 1.5 °C and 2.0 °C scenarios. A fictional datetime index was introduced for data generation between 2091 and 2101.

### Ensemble bias correction

Biases in temperature data were identified and corrected in the entire distribution through a quantile mapping approach^18^. To perform this operation, we re-gridded ERA5 reanalysis dataset^19,20^, with a spatial resolution of 0.25° for lat-lon grid, to our 0.833° × 0.556° grid. Biases are calculated for each percentile using the cumulative distribution functions from the differences between the historical model and ERA5 observations. The computed biases were then added to the historical 1.5 °C and 2.0 °C temperature scenarios in their corresponding percentiles. This method assumes that the bias is the same across scenarios.

The statistical approach for bias correction was applied to the combined ensemble, involving 70 individual members for a 10-year period, following a similar process to the ensemble bias correction method defined by Ayar *et al*.^21^. This approach ensures the preservation of the internal variability of a multi-member ensemble after the corrections.

Observations for bias correction are taken from ERA5^20^ for the same historical timeframe and spatiotemporal resolution used in this work’s historical scenario. ERA5 is the fifth-generation atmospheric reanalysis, produced by the Copernicus Climate Change Service (C3S) at the European Centre for Medium-Range Weather Forecasts (ECMWF). It provides hourly estimates at a 0.25° × 0.25° grid on the global climate and weather for the past four to seven decades. Data is available from 1940.

The bias correction was applied following a temporal cross-validation that divides the data period (10 years) into a set of independent validation periods. These independent periods are related to the season (or batch) with data between two and four months, following the same structure of raw generated data. For each historical batch, bias correction is performed in the entire ensemble, using temperature data from ERA5 for the same timeframe as a reference model. Then, the computed biases were added to the projected scenarios (batches) in their corresponding percentile. This ensures that the cumulative distribution of the historical ensemble matches the cumulative distribution of the observations (over the calibration period) and the preservation of the ensemble’s internal variability. The code for the bias correction method is available in the code availability section.

## Data Records

The data is available in the National Centre of Environmental Data under the Open Government Licence 3.0– CEDA^22^:

The dataset contains a 6-hour mean temperature output from the model experiment, resulting in 70 members for 10 years per scenario. As a result, each scenario includes 700 NetCDF V4 files (*.nc), one file per year. The horizontal resolution is 0.833° longitude and 0.556° latitude.

Table 1 summarises the structure of the data. There are three climate scenarios: historical (2006–16), 1.5 °C, and 2 °C above pre-industrial levels. These scenarios are divided into batches of data according to seasons between two or four months.

## Technical Validation

The temperature data published through this descriptor is a set of ensembles for the world’s land surface. For technical validation, the mechanisms used within the previous studies^23^ are valid here too.

**Fig. 1:**
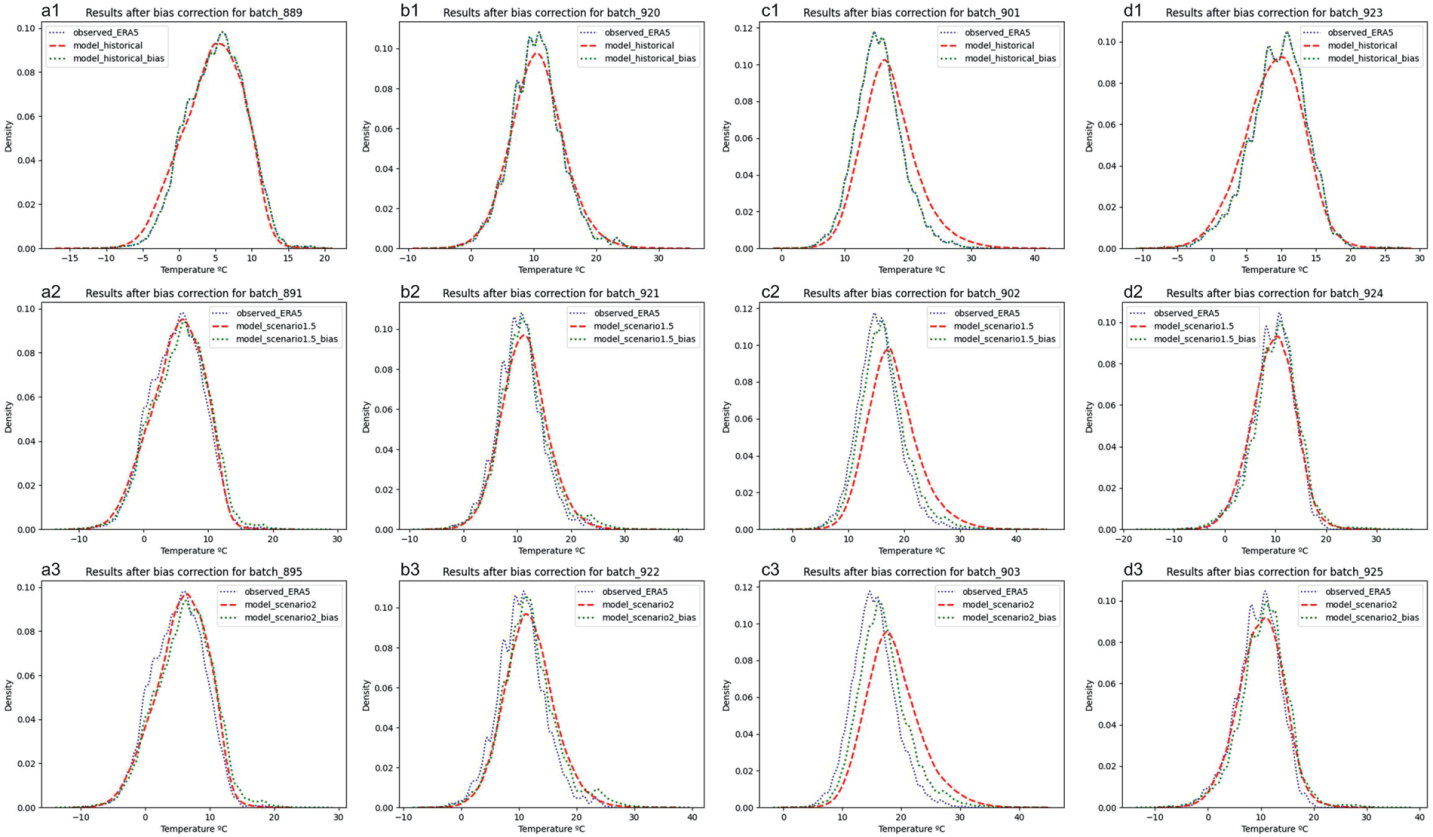
Data distribution of the entire ensemble before and after bias correction compared with ERA5. Data analysis was generated for a latitude and a longitude located in the UK: −1.7° longitude and 52.2° latitude. Note: in the legend, “model_*_bias” refers to data with bias corrected.

Figure 1 shows an example of the data distribution of simulated mean temperature compared with the observed ERA5 reanalysis dataset over the UK before and after bias correction. The simulations show agreement between the model and observations for the historical scenario (2006–16) across land surface data points (e.g. in Fig. a1-d1). The summer season for the Northern Hemisphere (Figs. 1, c1 - batch 901) presented a more intense bias, as noted by Watson *et al*.^11^, with higher differences between model distribution and observations. After bias correction (green dashed line), the historical ensemble model showed a good representation of the observed temperature data.

**Fig. 2:**
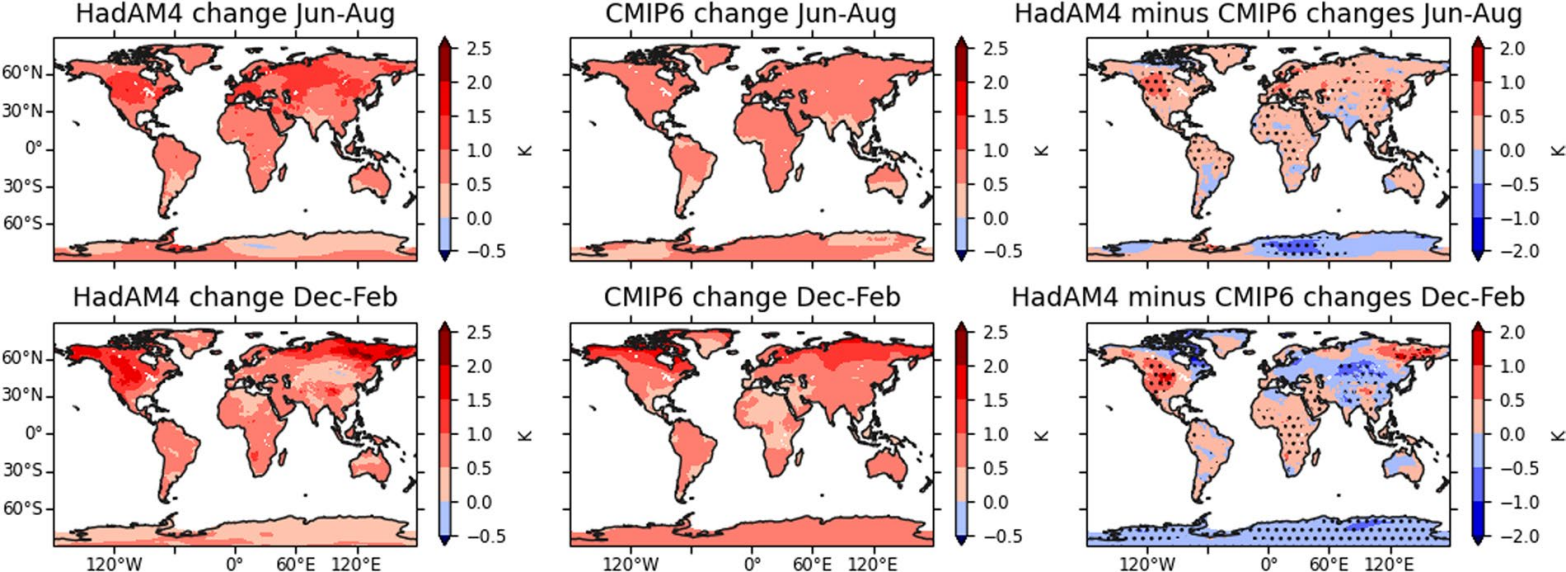
Changes in the mean surface air temperature between the 1.5 °C and 2 °C global warming scenarios. The left column shows the change in the HadAM4 simulations, and the middle column shows the multi-model change in the CMIP6 SSP2-4.5 scenario simulations between 1.5 °C and 2 °C warming levels. The right column shows the difference between these, with stippling indicating the statistical significance of differences at the 95% level based on sampling variability in the HadAM4 data (see text). The top row shows changes in the June-August season, and the bottom row shows changes for December-February.

The mean warming signal in the HadAM4 simulations after bias correction globally is compared with the multi-model mean in the Coupled Model Intercomparison Project Phase 6 (CMIP6) climate model simulations in Fig. 2. In particular, we present the change in temperatures from the 1.5 °C to the 2.0 °C scenario as these can be calculated directly from our results and this CMIP6 data recently reported by IPCC. The examination of this change in temperatures is policy-relevant, as a recent UN report suggests that the aspirational 1.5 °C limit set in the Paris Agreement is unlikely. Thus, there is a question on how increasing the global mean temperature limit from 1.5 °C to 2.0 °C will affect local temperatures.

An ensemble of 100 members of the HadAM4 simulations (10 from each year) is used, as this is sufficient to estimate the mean warming signal. We used surface air temperature data at 1.5 °C and 2 °C warming levels in CMIP6 simulations following the SSP2-4.5 scenario. Results using data at the same warming levels from SSP5-8.5 scenario runs are very similar (not shown), so they are not sensitive to the choice of climate scenario.

HadAM4 generally shows similar warming patterns to the CMIP6 multi-model mean, with mean temperature differences mostly between 0.5–1 °C and greater warming at high northern latitudes than at low latitudes in December-February (Fig. 2, left and middle columns).

The differences between the warming in HadAM4 and the CMIP6 mean in June-August are predominantly positive (Fig. 2, top right). Averaged over 30–90°N, HadAM4’s warming is 30% larger than the CMIP6 mean, and it is 15% larger between 30°S-30°N, though more similar in the southern extratropics. In December-February, HadAM4’s warming is 13% larger between 30°S-30°N but within about 2% in the extratropics (excluding Antarctica) (Fig. 2, bottom right). Therefore, the warming estimated based on HadAM4 will typically be slightly larger than if the CMIP6 multi-model mean climate response were used. However, these differences are within the range of differences seen between other models (e.g. Wehner *et al*.^24^) and are not clearly unrealistic.

Statistical significance of the differences is estimated by bootstrapping years of the HadAM4 simulations, with the mean difference from CMIP6 subtracted, and estimating the frequency with which mean differences from the CMIP6 mean in these samples exceed the magnitude of the actual differences between the HadAM4 and CMIP6 mean warming. This tests the null hypothesis that the HadAM4 and CMIP6 multi-model mean warming signals are the same, and the differences in Fig. 2 can be explained by interannual variability in the HadAM4 simulations. 200 bootstrap resamples are used. Note this does not account for sampling uncertainty in the CMIP6 mean, which would lower the statistical significance, so our analysis here will somewhat overstate the statistical significance of the differences between warming in the HadAM4 and the CMIP6 average. Based on this test, the aforementioned differences in area-averaged warming are statistically significant beyond the 99% level.

In terms of spatial pattern, the differences between HadAM4 and the CMIP6 multi-model mean warming (Fig. 2, right column) are mostly smaller than 0.5 °C. Grid points are stippled where fewer than 5% of the bootstrap resampled data have differences larger than those between HadAM4 and the CMIP6 mean. The main difference in the pattern between the HadAM4 and CMIP6 mean warming signal in June-August is that HadAM4 has greater warming by 0.5–1 °C in north western North America. This is also present in December-February, along with higher warming in northeast Asia and lower warming in central Asia. These differences in temperature response from the multi-model mean are similar to, or smaller than, those recorded between other climate models (e.g. Wehner *et al*.^24^).

## Usage Notes

A set of scripts is available to utilise the data generated as a cohesive whole. These are available from https://github.com/lizanafj/python_examples_with_NetCDF4_files and include examples with different NetCDF libraries to perform specific analyses across the dataset. Additional tools are available with NetCDF capabilities depending on user preference and aims. Tools for interaction with NetCDF are listed on a page maintained by the creators of the NetCDF standard, the Unidata program from UCAR (http://www.unidata.ucar.edu/software/netcdf/software.html).

## Data Availability

The code with the ensemble bias correction method using the quantile mapping approach is available at https://github.com/lizanafj/ensemble-bias-correction.
